# ST105 Lineage of MRSA: An Emerging Implication for Bloodstream Infection in the American and European Continents

**DOI:** 10.3390/antibiotics13090893

**Published:** 2024-09-18

**Authors:** Alice Slotfeldt Viana, Laís Pires do Valle Tótola, Agnes Marie Sá Figueiredo

**Affiliations:** 1 Departamento de Microbiologia Médica, Universidade Federal do Rio de Janeiro, Rio de Janeiro 21941-902, Brazil; alice.viana@micro.ufrj.br (A.S.V.); laispirestot@gmail.com (L.P.d.V.T.); 2 Faculdade de Medicina, Programa de Pós-Graduação em Patologia, Universidade Federal Fluminense, Niterói 24033-900, Brazil

**Keywords:** MRSA infection, hospital infection, CC5, clonal complex 5, methicillin-resistant *Stapylococcus aureus*

## Abstract

Sequence-type 5 (ST5) of methicillin-resistant *Staphylococcus aureus* (MRSA), harboring the staphylococcal chromosomal cassette *mec* type IV (SCC*mec*IV), was first detected in Portugal. It emerged as a significant cause of healthcare-associated (HA) infection in pediatric units and was hence named the pediatric clone. Another ST5 lineage, which carries SCC*mec*II, also prevailed in the USA and Japan for multiple years. More recently, another MRSA lineage, ST105-SCC*mec*II, part of the evolution of clonal complex 5 (CC5) MRSA, has emerged as the cause of hospital-acquired bloodstream infection outbreaks in countries including Portugal, the USA, and Brazil. This article reviews studies on the epidemiology and evolution of these newly emerging pathogens. To this end, a search of PUBMED from inception to 2024 was performed to find articles reporting the occurrence of ST105 MRSA in epidemiologic studies. A second search was performed to find studies on MRSA, CC5, ST5, and SCC*mec*II. A search of PUBMED from 1999 to 2024 was also performed to identify studies on the genomics and evolution of ST5, CC5, and ST105 MRSA. Further studies were identified by analyzing the references of the previously selected articles from PUBMED. Most articles on ST105 MRSA were included in this review. Only articles written in English were included. Furthermore, only studies that used a reliable genotyping method (e.g., whole genome sequencing, or MLST) to classify the CC5 lineages were selected. The quality and selection of articles were based on the consensus assessment of the three authors in independent evaluations. In conclusion, ST105-SCC*mec*II is an emerging MRSA in several countries, being the second/third most important CC5 lineage, with a relatively high frequency in bloodstream infections. Of concern is the increased mortality from BSI in patients older than 15 years and the higher prevalence of ST105-SCC*mec*II in the blood of patients older than 60 years reported in some studies.

## 1. Introduction

Methicillin-resistant *Staphylococcus aureus* (MRSA) poses a significant public health challenge due to its high morbidity and mortality rates, extended hospital stays, healthcare expenses, and limited therapeutic options for serious and disseminated infections [1]. The death toll attributed to MRSA infections is alarming. In 2019, a study spanning 204 countries and territories estimated that MRSA caused 100,000 deaths and 3.5 million DALYs (disability-adjusted life years) [2]. MRSA is a burden even in wealthier nations like the United States. According to the 2019 Antibiotic Resistance Threats Report from the Centers for Disease Control and Prevention [3], the estimated number of hospitalized MRSA patients reached 323,700 in 2017. This accounted for approximately 10,600 deaths and an estimated USD 1.7 billion in healthcare costs that year. Although the estimated number of cases has decreased from 2012 (401,000) to 2017, it is still very high [3]. Furthermore, the rate (cases per 100,000 US population) of invasive MRSA infection for 2020 is similar to the rate for 2017 (20.1 and 20.7, respectively) [4,5]. In Latin American countries, the percentage of MRSA in bloodstream infections (BSIs) was roughly between 40 and 60% of total *S. aureus* isolates [6,7].

A limited number of MRSA strains are responsible for both healthcare-associated (HA) and community-associated (CA) infections. The molecular epidemiology studies of MRSA typically rely on defining lineages or clones using multilocus sequence typing (MLST), which groups strains into sequence types (STs) and clonal complexes (CCs) (https://pubmlst.org/, accessed on 23 August 2024), and staphylococcal cassette chromosome typing (SCC*mec*), which groups strains based on variations in the SCC*mec* (https://cge.food.dtu.dk/services/SCCmecFinder/, accessed on 23 August 2024). MRSA strains within the same lineage (ST-SCC*mec*) often share many more features than just SCC*mec* type and ST type separately. These lineages may also share a preferred environment to cause infection [e.g., community (ST8(CC8)-SCC*mec*IV related to USA300) and hospital settings [ST239(CC8)-SCC*mec*III/related to Brazilian clone)] or a specific host [e.g., ST398(CC398)-SCC*mec*IV, associated with livestock animals (LA)]. Also, it is worth noting that MRSA lineages may share a similar antimicrobial resistance profile. For example, most ST239-SCC*mec*III strains display high-level multidrug resistance, while most ST8-SCC*mec*IV strains are more susceptible to non-ß-lactam antibiotics [8,9,10,11]. However, these barriers can be overcome. For example, CA-MRSA can cause HA infections [8], and livestock MRSA (LA-MRSA) can infect humans [12]. Bacteremia is often observed in HA infections, frequently caused by typical HA-MRSA strains, while CA-MRSA is commonly associated with skin/soft tissue infections (SSTI) and other non-bloodstream infections [11,13]. However, severe infections can occasionally be associated with CA-MRSA strains, including necrotizing pneumonia and necrotizing fasciitis caused by Panton–Valentine-producing MRSA [14].

MRSA was initially classified into clones based on PFGE (pulsed-field gel electrophoresis) patterns, named according to the geographic region of isolation such as the Brazilian clone (ST239-SCC*mec*III), New York/Japan clone (ST5(CC5)-SCC*mec*II), and Southwest Pacific clone (ST30(CC30)-SCC*mec*IV), or the locale they frequently caused infections, like the pediatric clone (ST5-SCC*mec*IV) [15,16,17,18]. However, the recent use of whole-genome sequencing (WGS) strategies and phylogenomic analysis has shown that MRSA isolates within the same MLST, SCC*mec* type, and even those sharing the same *spa* type (http://spaserver.ridom.de/, accessed on 23 August 2024) can cluster into unique phylogenetic clades [7,9,17,18]. Therefore, these should be regarded as separate clones. For instance, the CA-MRSA clone USA400 [ST1(CC1)-SCC*mec*IV] disseminated in the USA exhibits notable genetic and epidemiological differences compared to ST1-SCC*mec*IV MRSA strains observed in Brazil. ST1 from Brazil (ST1-BR) is associated with HA-MRSA infections and lacks *lukSF* genes for Panton–Valentine leucocidin (PVL), while USA400 is linked with CA-MRSA infections and carries *lukSF* genes. The genomes of USA400 and ST1-BR have been categorized into distinct phylogenetic clades, implying no direct relation [19]. Similarly, the CA-MRSA USA300 prevalent in North America (USA300-NA) belongs to a different phylogenetic clone than the USA300 variant (USA300-LA) disseminated in Colombia and other Latin American countries. These variants cluster in two dominant clades that vary by geographical region [20]. Hence, recent genomic epidemiology studies of MRSA suggest that WGS is the most precise technique for identifying MRSA-specific clones within the same lineage [9,11,19,20,21].

ST5-SCC*mec*II strains have spread beyond the borders of the USA and Japan, becoming more prevalent in various regions globally. The most common CC5 lineages are ST5-SCC*mec*II, associated with the New York/Japan clone, and ST5-SCC*mec*IV, linked to the pediatric clone [11,22,23,24]. The relatively recent diversification of ST105(CC5)-SCC*mec*II MRSA [21], its occurrence in different countries, its prevalence in bloodstream outbreaks in hospitals of industrialized countries, its extensive presence in BSI in the metropolitan area of Rio de Janeiro, Brazil [11], and the fact that its clinical importance may be underestimated due to the relatively expensive and time-consuming methods for MRSA genotyping [11,21] prompted us to write this review. In this article, we illustrate the global distribution of ST105-SCC*mec*II strains, highlight significant milestones in this MRSA strain’s genomic evolution, and discuss the possible threat this lineage presents to human health. Table 1 summarizes the most common MRSA lineages/clones, their molecular classification, and main geographical spread locations. 

## 2. The Clonal Complex 5

The clonal complex 5 (CC5) has an interesting historical trajectory. A study investigating the molecular chronology of CC5 strains in the Western Hemisphere estimated that the rise of this clonal complex began in the early 1960s/1970s, with the subsequent spread of ST5-SCC*mec*I in South America, particularly the Chilean-Cordobes clone. Conversely, ST5-SCC*mec*II spread throughout Central and North America [21]. ST5-SCC*mec*II MRSA, also known as the New York/Japan clone and USA100 clone, was first identified in the New York area in the late 1990s [18,25,26]. Later, a study examining 12 hospitals in seven US states revealed that the USA100 clone had spread beyond just New York [27]. In the early 2000s, the ST5-SCC*mec*II MRSA strains were also observed to be widespread in hospitals in Japan [28]. As a result, the clone initially named New York was renamed New York/Japan. Additionally, a systematic review of bacterial resistance within infections occurring in intensive care units (ICUs) highlighted the dominance of a few bacterial clones with multidrug-resistant phenotypes, with CC5-SCC*mec*II (USA100) still frequently circulating in US hospitals [29].

Since it was first detected in a Lisbon-based pediatric hospital in Portugal in 1992, strains of ST5-SCC*mec*IV (associated with the pediatric clone and USA800 clone) have circulated across various countries and continents [11,18,30,31,32]. This MRSA lineage is often found in young children, leading to its label as the pediatric clone [11,30,33,34]. Despite its capacity to provoke disseminated infections in adults, it is more repeatedly identified as a causative agent in infections in regions beyond the bloodstream, according to a study performed in Rio de Janeiro, Brazil [11]. However, in Spain, adult BSI was primarily attributed to CC5-SCC*mec*IV isolates [35].

In summary, these findings affirm that MRSA isolates in the same lineage can exhibit different epidemiological characteristics, despite having numerous shared genomic features. This implies that minor genomic alterations could potentially lead to differences in the epidemiological landscape.

In addition to the most commonly seen SCC*mec* types (II and IV), ST5 strains may also carry other SCC*mec* types, such as I and V [21]. For instance, a novel epidemic MRSA strain (ST5-SCC*mec*I) surfaced in Argentinean hospitals in 1999, replacing the prevailing Brazilian epidemic clone [BEC; ST239(CC8)-SCC*mec*III] [36]. In 2014, an outbreak of Staphylococcal Scalded Skin Syndrome in Italy was attributed to a rare MRSA clone from the ST5-SCC*mec*V lineage that produced the exfoliative toxin A (ETA), as stated by Lamanna et al. (2017) [37]. The occurrence of ST5 strains carrying diverse SCC*mec* types and subtypes strongly suggests the multiple SCC*mec* acquisitions by ST5 strains [38,39].

Interestingly, the first vancomycin-resistant *S. aureus* (VRSA) strain was found in Michigan, USA, in 2002 [40,41]. Since then, approximately 52 VRSA strains carrying *van* genes have been reported. Many of these isolates belong to CC5 [42,43,44]. In the USA, at least 13 of the 16 reported VRSA belong to this clonal complex. Although the prevalence of the CC5 background among VRSA strains is well documented, it is not yet understood why this is the case [21]. VRSA isolates are not solely confined to CC5; a VRSA strain from CC30 has also been detected in the USA [45].

Currently, CC5 MRSA is one of the most prevalent MRSA clonal complexes worldwide. A study executed in various South American countries showed CC5-SCC*mec*IV isolates as the second most common MRSA isolates from BSI, as stated by Di Gregorio et al. (2023) [46]. In a tertiary hospital in Kuwait, the leading clonal complexes were CC5 (31.6%) and CC6 (15.0%). Among the CC5, the most widespread were those containing SCC*mec*V, accounting for 52.6% [47]. Moreover, CC5 MRSA was the dominant clonal complex causing BSI in children at a hospital in Mozambique, Africa [48]. The ST764-SCC*mec*II lineage, a single-nucleotide variant of ST5, has been documented as a prevalent nosocomial pathogen in Asian countries, including China [49], Japan [50], and Thailand [51].

As previously mentioned, MRSA CC5 strains are typically associated with healthcare-related infections, frequently causing serious conditions in both adults and children. However, most of these strains fall under ST5-SCC*mec*II and ST5-SCC*mec*IV. The PubMed search using the terms “ST5 MRSA” revealed that the number of publications on this topic increased from 2003 to 2013 and then plateaued until 2023. This may reflect the stable presence of these MRSA as important hospital pathogens worldwide over the years.

While other STs have been detected among CC5, ST105 is particularly noteworthy, also being linked to serious disseminated infections [11,21,52,53,54,55,56]. In the MLST scheme, ST105 is a single nucleotide variant of ST5(CC5) characterized by a single mutation in the *yqiL* gene [17,57]. CC5 MRSA from different lineages may be involved in quite different epidemiological scenarios. ST5-SCC*mec*IV has a tropism for pediatric infections, whereas ST5-SCC*mec*II and ST105-SCC*mec*II are more involved in adult diseases. Indeed, it has also been found that CC5-SCC*mec*II strains, mainly ST105-SCC*mec*II, are more involved in BSI compared to ST5-SCC*mec*IV in some hospitals [11,30,31]. Another interesting set of data is related to the differences in antimicrobial resistance patterns. ST5-SCC*mec*II and ST105-SCC*mec*II isolates are often multidrug-resistant bacteria, whereas ST5-SCC*mec*IV are more susceptible to non-β-lactam antibiotics [11]. However, the molecular basis of these differences is not fully understood, and some aspects that have driven the evolutionary path of these CC5 lineages are presented in the Section 7.

## 3. Epidemiological History of ST105-SCC*mec*II

To the best of our knowledge, ST105-SCC*mec*II was initially reported in North America and Europe in the late 1990s [52,58]. From January to June 1998, 17 isolates of this lineage were recovered from various infectious samples, such as blood, urine, wounds, and the respiratory tract, in two different Miami hospitals. In these institutions, ST105-SCC*mec*II isolates ranked third in terms of frequency among MRSA lineages, trailing ST8(CC8)-SCC*mec*IV (associated with USA300) and ST36(CC30)-SCC*mec*II (associated with EMRSA-16). In Western Switzerland, ST105 isolates triggered MRSA outbreaks from 1999 to 2004. During this period, MRSA isolates (one isolate per patient) with an identical PFGE profile to that of ST105 isolates (termed clone B) were the predominant MRSA in the region, constituting 32% of all MRSA isolates. The initial outbreak induced by clone B occurred in a tertiary hospital that had enacted strict infection control measures. In 2001, another outbreak attributed to this clone was reported, affecting several healthcare centers in the region [58].

At the Milton S. Hershey Medical Center in Philadelphia, USA, 62 MRSA isolates were obtained from nasal swabs collected from patients admitted between 2008 and 2009. Of these, 22.4% were identified as ST105-SCC*mec*II, comprising the second most common clone identified in the hospital. The most frequent lineage found was ST5-SCC*mec*II/IV, accounting for 38.8% [59]. In a separate study conducted at Penn State Hershey Medical Center, a rural hospital in Pennsylvania, between August 2009 and March 2010, a total of 94 MRSA isolates were recovered from 151 case patients (62.25%). Of these isolates, 11 were typed as ST105-SCC*mec*II (11.70%), making it the third most prevalent lineage, following ST5 (34 isolates) and ST8 (26 isolates) [60]. Due to the small number of hospitals and MRSA isolates analyzed in Philadelphia, it is not possible to determine whether the observed difference in ST105 rate represents a true decrease in the ST105 incidence.

David and colleagues analyzed clinical MRSA isolates from various sources, including patients’ blood from different regions of Alaska. These isolates were collected between 2000 and 2006 at the Alaska Native Medical Center in Anchorage. Out of their collection of 224 MRSA isolates, 12 were classified as ST105, accounting for 5.4% of the total isolates [53]. ST105 is not only present in Alaska and Pennsylvania, but it is also found in other regions of the USA. ST105-SCC*mec*II has also been reported to cause severe and invasive outbreaks at Mont Sinai Hospital in New York [61].

Also, in a study involving seven hospitals in Brooklyn, New York, Iregui et al. (2019) [62] found that 14 out of 348 MRSA analyzed were ST105 (4.0%). In another study conducted in Iowa, USA, Fischer et al. (2020) [63] investigated the prevalence of various STs of *S. aureus* among patients with cystic fibrosis. Out of the 98 MRSA collected from 75 patients, ST5 was the most common MLST type (*n* = 54; 55.1%), followed by ST105 (*n* = 14; 14.3%). Similar results were obtained in a subsequent study by the same authors in 2021 with cystic fibrosis patients in Iowa [64].

ST105 MRSA has also been reported on the Pacific Coast of the USA. These isolates were acquired from clinical samples, such as blood, taken from inpatients at 30 different hospitals in Orange County, California, between October 2008 and April 2010. The ST105-SCC*mec*II lineage accounted for 4% of the 284 isolates that were selected for MLST typing; this made it the third most common, falling behind ST5 (45%) related to USA100 and ST8 (CC8) related to USA300 (38%) [65].

ST105-SCC*mec*II has also been identified in other European countries. In 2012, a study conducted across hospitals in Northern, Central, and Southern Italy analyzed 102 MRSA isolates. The results revealed that the USA100-like clone (ST5/105-SCC*mec*II) ranked as the fourth most prevalent MRSA strain; ST5 comprised 13 cases and ST105 accounted for just two cases [66]. ST105-SCC*mec*II was found at a low frequency in a collection of 83 MRSA isolates from BSI in Athens, Greece, from 2000 to 2015. During this period, the dominant lineages were ST239-SCC*mec*III and ST5-SCC*mec*II/IV, with only three (3.6%) ST105-SCC*mec*II isolates being recovered [67].

**Figure 1:**
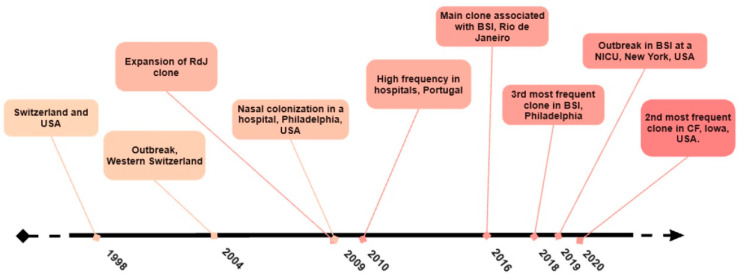
Timeline showing the major emergence events of strains of the ST105-SCC*mec*II lineage.

However, an increased prevalence of ST105 was observed in other European regions. Espadinha et al. analyzed the variations in the sample profile of MRSA obtained during two time periods, 1993 and 2010, and detected a clonal shift at a major tertiary teaching hospital in Portugal. By 2010, ST105 was found to be the second most frequent clone in the hospital (18.0%), trailing behind the dominant ST22-SCC*mec*IVh (72.0%) [68]. Furthermore, Faria et al. (2013) found that ST105-SCC*mec*II was the second most common MRSA in Portuguese hospitals, accounting for 19.0% [69]. Figure 1 provides a timeline summary of the major emergence events associated with ST105 MRSA strains.

A large cross-sectional study was conducted in Portugal to evaluate the carriage of *S. aureus* and MRSA between April 2010 and December 2012. This study involved a total of 3361 adults aged over 60 and examined both nasopharyngeal and oropharyngeal carriage of *S. aureus*. The results indicated the prevalence of ST105 MRSA. Most of the MRSA detected (82.3%) were related to three common HA-MRSA clones in Portugal: ST105-SCC*mec*II (43.5%), ST5-SCC*mec*IV (19.4%), and ST22-SCC*mec*IV (EMRSA-15 clone; 19.4%) [70]. The ST105 strain was also discovered in 2014 in Portugal, associated with SSTI, and was the main clone in this study (*n* = 7/34; 20.6%), followed by ST22 (*n* = 5/34; 14.7%) [71].

In 2010, a single ST105 MRSA isolate was reported in São Tome and Principe, Africa, from a colonization sample collected at Dr. Ayres Menezes Hospital. It has been suggested this isolate may originate from Portugal, a country where this clone is prevalent and maintains a demographic relationship with São Tome and Principe through tourism and patient transfers [72]. Evidence of international spread of isolates of the ST105 lineage has also been reported in genomic studies, where directly related strains from the USA and Brazil were grouped in the same phylogenetic clade [11].

A study conducted in Kuwait, Western Asia, analyzed 400 MRSA isolates from clinical samples collected between 1992 and 2010 from 13 hospitals. The study found the ST105-SCC*mec*II-t002 MRSA was first detected in 2010 at a low frequency of 0.5% [73]. Among a HA-MRSA group analyzed between 2012 and 2017 in China, only three isolates of ST105-SCC*mec*II-t688 (representing 2.5%) were found. The majority of the identified MRSA was ST239-SCC*mec*III (at 61.7%) [74]. These data suggest that ST105 is still uncommon in some continents, including Asia.

ST105 MRSA has been detected in both Central and South America. Studies analyzing 386 MRSA genomes from the American continent, collected from BSI samples from 2011 to 2018, revealed that ST105 isolates represented 17.9% of those samples [75]. In a separate prospective study that was carried out in nine Latin American countries between January 2011 and July 2014 and included *S. aureus* strains, it was discovered that the ST5-SCC*mec*II strain was widespread (>80%) in Mexico, Guatemala, and Brazil. However, in Colombia and Ecuador, over 70% of MRSA strains were classified as USA300-LV/LAE, demonstrating a diversity of strains present in the region. Out of the 96 isolates that were subjected to WGS, ST105 accounted for approximately 10% and was found in Brazil, Chile, and Colombia [6].

In a study conducted on *S. aureus* isolates collected between 2005 and 2013 from various clinical settings in Quito, Ecuador, it was reported that two out of 62 MRSA isolates were ST105 [76]. Martínez and colleagues used WGS to analyze 469 MRSA isolates obtained from Chilean hospitals between 1999 and 2018. They found the most widespread clone was ST5-SCC*mec*I, accounting for 70.1%, followed by ST105-SCC*mec*II at 14.9% [55].

While ST105-SCC*mec*II is not commonly found in certain Latin American countries, it was present in a study conducted in a São Paulo hospital from August 2010 to January 2012. The study identified nine clones of MRSA from nasal and groin swabs taken from 190 pre- and post-liver transplant patients. Out of the 69 MRSA isolates identified, 25, equivalent to 36.2%, belonged to the predominant clone, ST105-SCC*mec*II [77].

**Figure 2:**
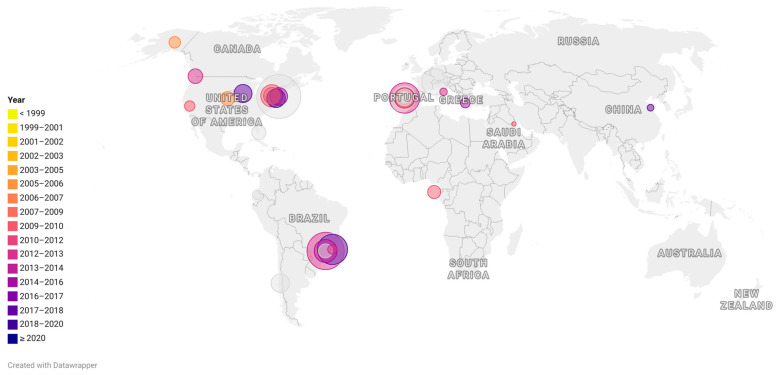
Representative map showing the occurrence of Sequence-type (ST)105 around the world. Each circle represents a scientific article in which ST105 was reported. The size of the circles indicates the percentage of ST105 reported among the collection included in each study. The colors indicate the date (in years) of the sample collection. For studies performed in a large period of years, the last year of the reported period was considered.

It is noteworthy that USA300 and EMRSA-15 isolates, which were found at higher frequencies in hospitals in the USA and Portugal, respectively, compared to ST105 strains, were also common causes of community-acquired infections, whereas ST105 strains are typically HA-MRSA. In addition, both USA300 and EMRSA-15 are generally more susceptible to non-beta-lactam antibiotics than ST105 [11,20,21,53,70,71]. This suggests that resistance characteristics alone cannot explain the prevalence of some MRSA lineages. However, the molecular mechanisms involved in the prevalence of specific MRSA lineages remain to be elucidated [19]. Figure 2 illustrates the prevalence of ST105 MRSA in different countries.

## 4. ST105-SCC*mec*II in Bloodstream Infections

A 2019 study estimated that among 33 bacterial pathogens, just five leading pathogens were responsible for 56.2% of all sepsis-related deaths. *S. aureus* was the leading cause of death in 135 countries. The most deadly infectious syndrome and pathogen were associated with patient region and age, and the majority of deaths occurred in patients older than 15 years. In 2019, more than 6 million deaths were caused by three types of bacterial infections, with BSI and lower respiratory tract infections dominating with more than 2 million deaths each, followed by perineal and intra-abdominal infections, which caused more than 1 million deaths [78]. In another global study, *S. aureus* was second only to *Escherichia coli* among the top six antimicrobial-resistant pathogens, each causing approximately 50,000 to 100,000 deaths [2]. These data underscore the importance of MRSA in serious and life-threatening infections such as BSI.

Among MRSA, CC5 isolates, mainly ST5-SCC*mec*II, have been described in high frequency in BSI in different hospitals worldwide, and it was reported with a higher global frequency of 68% in a large medical center in Minnesota, USA [46,79,80]. As in this Minnesota study, the emerging ST105-SCC*mec*II has been reported as a cause of BSI with variable frequency in different European and American hospitals, generally being the second most common MRSA lineage after ST5-SCC*mec*II [6,11,54,55,61,69,75,81,82]. Read and colleagues analyzed 105 MRSA isolates from bloodstream infection cases in two hospitals in Philadelphia, Pennsylvania, between July 2018 and June 2019, using WGS. Of the 105 isolates, 16 belonged to ST105 (15.84%). The majority of the MRSA isolates were classified as CC8 (*n* = 55), with USA300 (*n* = 49) being the dominant strain, followed by CC5 *(n* = 40). ST105 was the second most common CC5 lineage [54].

Berbel Caban and collaborators (2020) traced an “under-the-radar” outbreak caused by ST105-SCC*mec*II-t002 involving 16 patients with bacteremia and two hospitals in New York [83]. Another survey of 132 MRSA genomes from blood isolates showed that a multi-month outbreak in a neonatal intensive care unit (NICU) at Mont Sinai Hospital in New York was caused by ST105-SCCmecII isolates. Hospital-wide genomic surveillance data traced the origin of the outbreak to 3 patients admitted to adult wards 4 months before the NICU outbreak. Genomic studies show that the ST105 strain responsible for the outbreak had unique mutations and genetic elements that affected genes related to metabolism, resistance, and persistence. Transcriptome sequencing (RNA-Seq) profiling revealed that epigenetic changes in the outbreak clone repressed *agr* gene expression and upregulated genes associated with stress response and biofilm formation [61]. Furthermore, Faria et al. (2013), focusing exclusively on isolates from bloodstream infections in Portuguese hospitals, identified two predominant MRSA clones: EMRSA-15, representing 75.0% of MRSA isolates, and ST105-SCC*mec*II, accounting for 19.0% [69].

Studies analyzing 386 MRSA genomes from the American continent, collected from BSI samples between 2011 and 2018, found that ST105 isolates represented 17.9% of these samples [75]. A large epidemiological study of 600 MRSA isolates from 51 hospitals in the Rio de Janeiro metropolitan area showed that ST105-SCC*mec*II was the most prevalent among BSI isolates. Of the 245 MRSA isolates from BSI, 115 (46.9%) were CC5-SCC*mec*II, and 73.2% of these were ST105-SCC*mec*II. Moreover, ST105-SCC*mec*II was shown to have a greater ability to evade phagocytosis compared to other CC5-SCC*mec*II/IV clones. As suggested by the authors, this may explain its higher prevalence in BSI [11]. Additionally, an independent study conducted at a university hospital in Rio de Janeiro documented that the majority of blood isolates (43.2%) were ST105-SCC*mec*II [81]. Viana et al. found a higher frequency of this lineage in patients older than 60 years compared to other MRSA lineages, such as ST5-SCC*mec*IV and ST30-SCC*mec*IV [11]. Also in Portugal, Almeida and coworkers found an increased isolation of ST105-SCC*mec*II in BSI in patients older than 60 years [70].

## 5. ST105-SCC*mec*II and Its Dissemination among Animals and the Environment

MRSA has been identified in domestic animals. A study conducted in 2006 explored the diversity of MRSA isolates within veterinary clinics in the Midwest and Northeastern areas of the USA. From 24 isolates, approximately half were found to match the PFGE profile USA100, correlated with clones ST5 and ST105. The study also reported the existence of other human MRSA clones, thus suggesting potential transmission from humans to animals [84].

Couto et al., in Portugal, found four strains that belonged to CC5 among animal isolates, one of which was of canine origin and belonged to ST105-SCC*mec*II [85]. The ST105-SCC*mec*II lineage has been identified as a nasal colonizer of MRSA in individuals in Portugal who are in regular contact with animals. Moreover, the study also detected the presence of the primary human healthcare clones in Portugal, including ST22-SCC*mec*IV and ST105-SCC*mec*II, along with LA-MRSA ST398-SCC*mec*IV [86].

In 2014, it was discovered that two urban Norway rats in Vancouver’s Downtown Eastside neighborhood were colonized by ST105 MRSA. In addition to ST105, these rats were also colonized by other clones, such as ST97 and ST398, which are frequently found in both human and livestock populations [87]. More recently, an ST105-SCC*mec*II isolate was identified among 16 MRSA samples taken from purulent lesions in rabbits at a Portuguese slaughterhouse [88].

Hospital wastewater often contains antibiotic-resistant bacteria, including MRSA. A study conducted with waste samples from three hospitals in northern Portugal detected MRSA isolates belonging to ST22-SCC*mec*IV, ST8-SCC*mec*IV, and ST105-SCC*mec*II. These findings highlight the capacity of these lineages to survive in untreated hospital effluents [89]. The environmental discharge of MRSA, including ST105-SCC*mec*II, brings about its detection in rats. Given the promiscuity of gene transfer in bacteria, the presence of multidrug-resistant MRSA such as ST105 isolates in the environment should be considered an ecological concern. Furthermore, the environmental spread of ST105-SCC*mec*II may reflect a higher frequency of this lineage in humans than has actually been reported. Table 2 shows the occurrence of ST105 MRSA in humans and animals from different countries.

## 6. Antibiotic-Resistance Pattern of ST105-SCC*mec*II

In 2007, Mwangi et al. conducted a study exploring the mechanisms of vancomycin resistance. Isogenic *S. aureus* isolates were obtained from a patient’s bloodstream both before the commencement of vancomycin treatment and after the failure of the said treatment. The initial isolate, identified as JH1 [GenBank Accession number (Acc): NC_009632], showed susceptibility to vancomycin, while the final isolate, JH9 (Acc: GCA_000016805.1), had developed resistance to it. Both isolates were categorized as ST105-SCC*mec*II and displayed a parallel 100-fold decrease in susceptibility to daptomycin [95]. Daptomycin, a cationic antimicrobial agent, coupled with vancomycin, is considered a last resort for treating MRSA BSI [96].

There have also been reports of daptomycin-resistant ST105 isolates due to mutations in various genes related to phospholipid syntheses and metabolisms, such as *mprF* (multiple peptide resistance factors), *cls* (cardiolipin synthesis), and *pgsA* (phospholipid metabolism). Further, alterations in regulatory genes *yycF/yycG* and *vraSR*, related to cell membrane stress and permeability, have been implicated too. Additionally, the transcriptional regulator *dltABCD*’s upregulation, which boosts the surface positive charge through teichoic acid D-alanination as seen with *mprF* mutations, has been linked to nonsusceptibility to daptomycin [97]. However, the overall impact of this resistance profile among ST105 isolates worldwide remains undetermined.

According to a study by Melo-Cristino et al. (2013), the first case of vancomycin-resistant *S. aureus* (VRSA) identified in Portugal is linked to the clonal background ST105-SCC*mec*II [98]. Notably, the strain FCFHV36 (Acc: CP011147), reported in 2015 as an MRSA strain heterogeneously intermediate to vancomycin, was recovered from a vertebral biopsy of an osteomyelitis patient at a hospital in Santa Catarina, Brazil, and belongs to the ST105-SCC*mec*II lineage [99]. These studies suggest that ST105 can acquire not only the *vanA* gene but also increase the minimum inhibitory concentrations (MICs) of vancomycin.

Silva et al. (2020) examined the antimicrobial resistance profiles of 16 bloodstream infection (BSI) isolates from Portugal. Among these, ST105-SCC*mec*II isolates exhibited resistance to quinolones and an inducible macrolide-lincosamide-streptogramin B (iMLS_B_) phenotype [94].

Iregui et al. (2020) investigated delafloxacin-resistant *S. aureus* isolates in seven hospitals in Brooklyn, NY (USA) [62]. They found that 14 of the 16 delafloxacin-resistant isolates selected for MLST were ST105-MRSA, and one was ST105-MSSA (MSSA: methicillin-susceptible *Staphylococcus aureus*). Analysis of these isolates revealed that all were found in patients with extensive healthcare system contact, and the resistance was due to an accumulation of multiple mutations in gyrase and topoisomerase IV genes. Viana et al. (2022) showed through genomic research involving 82 ST105 isolates from 2014–2016 in Rio de Janeiro, that 100% of ST105-SCC*mec*II isolates were aminoglycoside-resistant [mediated by the genes *aad*, *aph*(3′), or *ant*(9)]; 98.7% (*n* = 81/82) to macrolides [*erm*(A), *erm*(C), *msr*(A), or *mph*(C)] and 50% (*n* = 41/82) were resistant to chloramphenicol [*cat*(pC221) gene] [100]. It is concerning that all tigecycline-resistant isolates collected from ICU patients in Brazil over 6 months were identified as ST105 MRSA (*n* = 10/36; 27.8%) [101].

ST105-SCC*mec*II isolates from Rio de Janeiro have been discovered to have low-level triclosan resistance, which is facilitated by the Tn*Sha1* transposon [100,102]. Although resistance to low-dose triclosan is unlikely to affect the biocide’s effectiveness because it is typically used in high concentrations, it could potentially lead to complete resistance and the emergence of antimicrobial persisters [103].

## 7. Evolution of ST105-SCC*mec*II-t002

Challagundla et al. (2018) analyzed the genomic sequence of 598 CC5 isolates from various countries in the Western Hemisphere [21]. According to their studies, the most basal group, termed CC5-Basal, was predominantly represented by ST5-SCC*mec*IV and ST5 MSSA. This group also includes the genome of strain N315, which was previously reported to be related to the USA100/New York Japan clone, suggesting that strains related to N315 are not directly related with other NY/Japan-related genomes, which cluster in a different phylogenetic group. In fact, it was even proposed that the NY/Japan clone consists of two independent clones of the ST5-SCC*mec*II lineage [21]. The second group identified by these authors, the CC5-SCC*mec*I (CC5-I), likely emerged in the early 1970s. Its earliest branches represent isolates from Europe, with its expansion in South America occurring in the mid-1990s. CC5-I groups Chilean/Cordobes (ST5-SCC*mec*I) and South German (ST228-SCC*mec*I) clones, representing one of the three presumed acquisitions of SCC*mec*I by CC5 strains (the other two instances occurred in the CC5-Basal and CC5-IIB groups [21].

Strains from the CC5-II group predominantly hail from Central and North America, with some from South American countries like Brazil, Venezuela, and Ecuador. The CC5-II bifurcates into two subgroups: the paraphyletic basal CC5-IIA and the monophyletic terminal CC5-IIB, both of which encompass CC5-SCC*mec*II-carrying isolates procured at the onset of clade formation. Both subgroups contain USA100-related reference genomes (associated with the NY clone). This suggests that MRSA isolates from the NY clone are likely polyphyletic and have therefore accumulated mutations in their independent evolution [21]. This finding further bolsters the capacity of CC5 to adapt and evolve into multiple successful clones [11,21,54,55,75]. The CC5-IIA represents the early branches of ST5-II genomes from the USA and boasts a notable subclade with the ST228-II derivative of ST5 isolated in Mexico. The CC5-IIB terminal subclades signify the expansion of ST5-SCC*mec*II and the emergence of ST105-SCC*mec*II, ST225-SCC*mec*II, ST231-SCC*mec*II, and ST496-SCC*mec*II, isolated in both South America (specifically Chile, Brazil, and Ecuador) and North America (USA and Canada), with subsequent expansion in the USA. A distinct feature of the CC5-IIB clade is the loss of the *sep* gene encoding the staphylococcal enterotoxin P [21]. According to molecular chronology studies, it is estimated that ST105-SCC*mec*II originated in the early 1980s, several years after the specialization of ST5-SCC*mec*II [11,21]. Table 3 describes the major events in the evolution of clonal complex 5.

Viana et al. (2021) reported a significant spread of ST105-SCC*mec*II in hospitals throughout Rio de Janeiro [11]. These MRSA isolates, grouped into the terminal subclade of the CC5-IIB clade, became known as the Rio de Janeiro (RdJ) clone. In addition, they discovered that during the evolution of CC5-SCC*mec*II, ST105 isolates not only lost *sep* but also lacked the *splD* gene encoding for serine protease D. This omission persisted during the ST105-SCC*mec*II expansion, implying an evolutionary benefit for this lineage. Notably, the ST105 isolates from Rio de Janeiro demonstrated greater evasion of phagocytosis by THP-1 monocytes compared to the ST5-SCC*mec*II and ST5-SCC*mec*IV isolates, possibly explaining their higher prevalence in BSI [11]. Concurrent studies by these researchers suggest the loss of *splD* might have affected ST105 phagocytosis [105]. Pangenomic studies identified a specific mutation in the *aur* gene, which encodes the protease aureolysin, only in the RdJ clone. This protease contributes significantly to bacterial virulence, or ability to cause disease, by cleaving host factors of the innate immune system. The nonsynonymous mutation A1106G found in RdJ isolates was located in the recognition site of *aur*. However, the role played by this mutation in RdJ virulence remains to be elucidated [104].

Arias et al. (2017) detected the presence of ST105-SCC*mec*II in Brazil and Chile as well [6]. However, the Chilean ST105-SCC*mec*II strains described by Arias lack *aph*(39)-III, *msrA*, *mphC* (genes responsible for antimicrobial resistance), and the *chp* gene (from the immune evasion cluster; IEC). This is different from the recently described RdJ clone and other Brazilian ST5 strains, which carried these genes [6,11,55]. This observation suggests that the Chilean and RdJ clones present different genomic traits, despite belonging to the same lineage, ST105-SCC*mec*II. Once again, this demonstrates the great plasticity of these MRSA. In fact, pangenomic studies of CC30, CC5, and CC8 revealed that CC5 has the highest genetic variability. In addition, multiple virulence and resistance genes were identified, highlighting the complex virulence profiles of these MRSA strains [106].

Sullivan and colleagues (2019) analyzed the genome of ST105 strains involved in a BSI outbreak in a neonatal ICU in New York City [61]. They found that the outbreak strains had mutations in genes associated with bacterial metabolism, resistance, and persistence. Furthermore, they discovered a recombination present in the DNA recognition domain of the *hsdS* gene of the type I restriction-modification (R-M) system that had altered the DNA methylation. The study’s findings revealed that the transcriptome sequencing profile exhibited epigenetic alterations in the outbreak clone, an attenuation of *agr* expression, and an upregulation of genes governing stress response and biofilm development. It has been previously suggested that *agr* attenuation reduces bacterial cytotoxicity and is thus related to the bacterium’s ability to cause persistent bloodstream infections and disseminate within the hospital setting [107,108,109].

Altogether, these studies imply that ST105 strains not only have a high propensity to acquire resistance genes but can also adapt effectively to the human host to modulate their virulence and elude the immune system. These evolutionary adaptations may be attributable to mechanisms that can augment biofilm accumulation and stress response, for example, and reduce bacterial cytotoxicity. This allows the bacteria to evade the immune system and escape phagocytosis through mechanisms requiring further elucidation [11,21,61]. Moreover, the observation that ST105 is prevalent in blood infections also mandates further exploration, with the need to investigate the mechanisms implicated in this prevalence.

## 8. Conclusions

ST105-SCC*mec*II is an emerging MRSA lineage in European and American hospitals. Among CC5 isolates, it is often the second most important MRSA lineage, with a relatively high frequency in bloodstream infections in some countries. Moreover, the increased mortality from BSI in patients older than 15 years and the high prevalence of ST105-SCC*mec*II in the blood of patients older than 60 years should be of great concern.

## 9. Note of Authors

With the coronavirus disease 2019 (COVID-19) pandemic, the ST105-SCC*mec*II in Rio de Janeiro has drastically dropped in bloodstream infections, with the rise of both North and Latin American USA300 variants, although RdJ are still frequent in BSI affecting mainly old people [110].

## Figures and Tables

**Table 1:** Methicillin-resistant *Staphylococcus aureus* lineages/clones common names and their most frequent geographical spread location.

MLST-SCC*mec*	Common Name	Other Name	Geographical Spread *
(CC5)ST5-SCC*mec*I	ChC/EMRSA-3	Chilean-Cordobes clone/Epidemic MRSA-3	South America/Europe
(CC5)ST5-SCC*mec*II	USA100	New York/Japan clone	All continents
(CC5)ST5-SCC*mec*IV	USA800	Pediatric clone	All continents
(CC5)ST225-SCC*mec*I	-	South German clone	Europe
(CC8)ST8-SCC*mec*IV-PVL-	USA500	-	USA
(CC8)ST8-SCC*mec*IV-ACME+/COMER+	USA300	USA300-NAE/USA300-LAE	Europe, North America/South America
(CC8)ST239-SCC*mec*III	BEC	Brazilian epidemic clone, Hungarian clone	South America, Europe, Asia
(CC30)ST30-SCC*mec*IV	USA1100	Southwest Pacific clone	Oceania, South America, Europe
(CC30)ST36-SCC*mec*II	USA200 (EMRSA-16)	-	Europe

* Main geographic regions where these clones were spread [6,8,20].

**Table 2:** Articles reporting the occurrence methicillin-resistant *Staphylococcus aureus* belonging to the sequence-type (ST) 105 from human and animal sources.

Author, Year	Geographic Region	Population Studied	ST105 *n* (%)
Almeida et al. (2015) [70]	Portugal	One healthcare center/colonization in patients ˃60 years old	27 (43.5%)
Blanc et al. (2007) [58]	Western Switzerland	One reference laboratory/clinical isolates	655 (32.0%)
Viana et al. (2021) [11]	Rio de Janeiro, Brazil	51 hospitals/colonization and nosocomial infections	82/179 (45.8%)
Verghese et al. (2012) [59]	Pennsylvania, USA	One hospital/colonization	15 (22.5%)
Faria et al. (2013) [69]	Portugal	12 hospitals/BSI	18 (18.%)
Espadinha et al. (2013) [68]	Portugal	One hospital/nosocomial infections and nine healthcare centers/community SSTI	30 (16.5%)
Read et al. (2020) [54]	Philadelphia, USA	Two hospitals/BSI	16 (15%)
Sullivan et al. (2019) [61]	New York, USA	One hospital/BSI	18 (13.5%)
Peterson et al. (2012) [60]	Pennsylvania, USA	One hospital/colonization	11 (11.7%)
Himsworth et al. (2014) [87]	Vancouver, Canada	Downtown Eastside (DTES) neighborhood/colonization in rats	2 (9.1%)
Lin et al. (2011) [84]	USA	Six veterinarian settings/clinical isolates	2 (8.3%)
Chung et al. (2004) [52]	Miami, USA	Two hospitals/clinical isolates	17 (8.4%)
Conceição et al. (2014) [72]	São Tomé e Príncipe	One hospital/colonization	1 (7.1%)
David et al. (2012) [53]	Alaska, USA	One reference laboratory/clinical isolates	12 (5.3%)
Hudson et al. (2013) [65]	California, USA	30 hospitals/clinical isolates	12 (4.0%)
Nikolaras et al. (2019) [67]	Athens, Greece	One hospital/BSI	3 (3.6%)
Campanile et al. (2015) [66]	Italy	52 hospitals/nosocomial infections	2 (1.9%)
Peng et al. (2018) [74]	Shandong, China	One hospital/clinical isolates	3 (1.5%)
Boswihi et al. (2016) [73]	Kuwait	13 hospitals/clinical isolates and colonization	2 (0.5%)
Zuma et al. (2017) [90]	Rio de Janeiro, Brazil	Five hospitals/BSI	2 (3.3%)
Okado et al. (2016) [91]	São Paulo, Brazil	One hospital/patients with HIV	7 (24.1%)
Arias et al. (2017) [6]	Latin America	24 hospitals/BSI	8 (8.3%)
Porterfield et al. (2021) [64]	Iowa, USA	One hospital/patient with cystic fibrosis	14 (14.4%)
Couto et al. (2015) [85]	Portugal	One hospital/BSI	2 (12.5%)
Silva et al. (2020) [82]	Portugal	Slaughterhouse/purulent lesions of rabbits	1 (6.25%)
Rodrigues et al. (2017) [86]	Portugal	27 Veterinarian settings/staff colonization	1 (5.2%)
Caiaffa-Filho et al. (2013) [92]	São Paulo, Brazil	One hospital/clinical isolates	4 (66.6%)
Zurita et al. (2016) [76]	Quito, Ecuador	Three hospitals/clinical isolates	2 (3.2%)
Ferreira et al. (2021) [93]	Lisbon, Portugal	One community laboratory/SSTIs	7 (20.5%)
Fischer et al. (2020) [63]	USA	One hospital/patients with cystic fibrosis	14 (14.8%)
Martinez et al. (2023) [55]	Chile	One hospital/clinical isolates	119 (14.9%)
Iregui et al. (2020) [62]	Brooklyn, New York	Seven hospitals/delafloxacin-resistant isolates	15 (93.7%)
Salgueiro et al. (2020) [94]	Portugal	Isolates sent to a reference laboratory/community and nosocomial infections	14 (24%)
Augusto et al. (2022) [81]	Rio de Janeiro, Brazil	One hospital/BSI	16 (43.2%)
Silva et al. (2020) [88]	Portugal	Slaughterhouse/purulent lesions of rabbits	1 (6.25%)

BSI = bloodstream infections; ST = sequence type; USA = United States of America; SSTI = skin and soft tissue infection.

**Table 3:** Evolutionary events and acquisition of virulence and antimicrobial resistance traits along the evolution of clonal complex 5.

Clade	Evolutionary Events	Virulence	Antimicrobial Resistance
CC5-Basal	Acquisition of SCC*mec*IVc/SCC*mec*I/SCC*mec*II/SCC*mec*III	Acquisition of *lukSF*-PVL, *sec*, *sel* and *etb*; sporadic acquisition of *tst*	Resistance to penicillin, sporadic vancomycin resistance; independent acquisition of low-dose resistance to triclosan
CC5-I	Acquisition of SCC*mec*I, diversification in ST228, ST111, ST1481	Loss of *fnbB*	Resistance to fluoroquinolones, macrolides, lincosamides and aminoglycosides
CC5-IIA	Acquisition of SCC*mec*II, origin of the New York USA100 clone, diversification in ST1011	Loss of *sep* and acquisition of *fnbB*	Multiple independent acquisition of vancomycin resistance; independent acquisition of low-dose resistance to triclosan
CC5-IIB	Diversification in ST1011, ST225, ST105, ST125, ST231 and ST496	Acquisition of *sed*, *sej* and *ser*; loss of *splD*; acquisition of *aur* mutation (A1106G) by RdJ clone.	Independent acquisition of low-dose resistance to triclosan

Phylogenetic groups were defined by Challagundla et al. (2018) [21]. The data presented in this table are a summary of previously reported major genomic events of CC5 MRSA [11,21,61,100,104].
